# Cadaveric Analysis of Accessory Foramina in the Orbito-Sphenoid Region

**DOI:** 10.7759/cureus.110482

**Published:** 2026-06-08

**Authors:** Dominic Venardi, Sunny Patel, Rachel Gutkin, Michael Cramberg, Sumitra Miriyala

**Affiliations:** 1 Anatomy, A.T. Still University - Kirksville College of Osteopathic Medicine, Kirksville, USA

**Keywords:** accessory foramina, cranial variations, neurovascular structures, orbito-sphenoid foramen, pre-surgical visualization

## Abstract

Purpose: This study confirms the presence of accessory foramina in the orbito-sphenoid region, reinforcing their potential impact on neurovascular injury and surgical outcomes. Continued data collection will enhance accuracy and generalizability, emphasizing the need for presurgical visualization to optimize patient safety and surgical success.

Methods: This study examined variations in the orbito-sphenoidal region using donor skulls (n=10) from A.T. Still University’s Gift of Body Program. Careful dissection exposed the optic canal and superior orbital fissure, and cranial variations were analyzed.

Results: Three donor skulls exhibited accessory foramina: two with bilateral accessory foramina and one with a single foramen in the left orbit. Foramina diameters ranged from 0.687 mm to 1.204 mm, with a mean of 1.015 mm. These findings suggest that cranial remodeling often results in variations such as accessory foramina, emphasizing the importance of preoperative visualization to reduce the risk of neurovascular damage. Further research in larger, more diverse populations is needed to better understand the clinical implications of these variations.

Conclusion: This study confirms the presence of accessory foramina in the orbito-sphenoid region, reinforcing the need for presurgical visualization to minimize neurovascular risks. Continued research will improve accuracy and generalizability, aiding in safer surgical planning.

## Introduction

The sphenoid bone is a central component of the skull that connects the cranium to the facial skeleton. This wedge-shaped bone lies posterior to the frontal bone and anterior to the occipital bone [1]. The sphenoid consists of the greater sphenoidal wing, which contributes substantially to the lateral orbital wall, while the lesser wing extends anterolaterally to form a smaller portion of the posterior wall. In addition to articulating with both cranial and facial bones to provide stability, the sphenoid contains several openings that serve as passageways for neurovascular structures [2,3].

Within the sphenoid, the greater wing houses the foramen rotundum, foramen ovale, and foramen spinosum, while the lesser wing contains the optic canal. The superior orbital fissure lies between the two wings and, together with the optic canal, forms a major neurovascular path through the orbit [2]. The optic canal carries the optic nerve and ophthalmic artery. The superior orbital fissure transmits the oculomotor nerve (CN III), trochlear nerve (CN IV), ophthalmic division of the trigeminal nerve (V1), abducens nerve (CN VI), and the ophthalmic vein. These structures are involved in ocular movement, vision, and sensation, making their protection during surgical procedures critically important [1]. During embryonic development, variations in sphenoid ossification may lead to differences in morphology, including the presence or absence of foramina, bones, and ligaments [4]. Our study specifically focuses on the identification of accessory foramina within the orbit.

Knowledge of anatomy when performing surgical procedures is essential for promoting patient safety [5]. One study identified the petrosal part of the sphenoid bone as a critical landmark for locating the abducens nerve, helping to minimize the risk of nerve injury during endoscopic endonasal surgeries [6,7], particularly relevant given its long trajectory and thus greater potential for injury [7,8]. While a deep understanding of normal anatomy is important, it is equally important to be aware of potential anatomical variations, as unexpected variations could negatively impact surgical outcomes [8]. For example, another study cited variations in sphenoid sinus morphology involving protruding neurovasculature. Such variations could have devastating consequences, as iatrogenic damage to the internal carotid artery could be fatal, and injury to the optic nerve could result in permanent visual impairment [9]. More recent anatomical investigations have identified multiple accessory foramina on the sphenoid bone, including the foramen of Vesalius and the foramen of Arnold, which have prevalences of approximately 70% and 30%, respectively. These studies illustrate substantial variability in the presence of accessory foramina in the sphenoid bone [10]. Recognition of these openings may aid in preoperative planning to reduce the risk of neurovascular damage during cranial tumor resection and other skull base surgeries [11].

These findings underscore that accessory foramina serve as pathways for neurovascular structures or for the spread of disease, thereby directly affecting surgical outcomes in the orbit and skull [6,11]. Therefore, continued research into the presence and variability of accessory foramina within the orbit is essential for improving anatomical understanding and enhancing the safety and precision of surgical procedures.

This work was previously presented as a poster at the Interdisciplinary Biomedical Research Symposium on November 15, 2025.

## Materials and methods

All procedures in the current study were considered exempt by the A.T. Still University-Kirksville Institutional Review Board. Ten Midwest donor body skulls from the Gift of Body Program at A.T. Still University's Kirksville College of Osteopathic Medicine were analyzed for structural variations. Dissection was completed in the orbital and sphenoid regions to expose the optic canal and superior orbital fissure. The following exclusion criteria were applied to donor body skulls: broken or damaged during dissection or preservation, evidence of extensive trauma, and skulls affected by disease. Demographics, past medical history, and cause of death for these skulls were unavailable, preventing us from forming correlations with our study data.

Study methodology

For the dissection, an anterior approach to the orbit was used. The cadaver was positioned supine with the orbit facing anteriorly. Superficial skin incisions were made to carefully dissect the upper and lower eyelids.

Using a blunt probe, the lateral and palpebral parts of the orbital portion of the orbicularis oculi muscle were raised and reflected medially to expose the eyeball. Transecting incisions along the most distal part of all six extraocular muscles (superior rectus, inferior rectus, lateral rectus, medial rectus, and the superior and inferior oblique muscles) were made to reflect the ocular muscles and expose the optic nerve. The optic nerve was cut to free and remove the eyeball. For educational purposes, the rectus muscles were reflected to reveal and appreciate the neurovasculature.

For excarnation, the specimen head was manually defleshed to remove major soft tissues and air-dried briefly. It was then placed in a controlled colony of dermestid beetles (21-27°C, low humidity) and left until all remaining soft tissue was removed. The cleaned skull was retrieved, degreased in a mild detergent or dilute ammonia solution, and rinsed. Final whitening was performed using hydrogen peroxide, followed by air drying prior to storage and analysis.

Each skull was then visually inspected for any abnormalities localized to the orbito-sphenoid region. Each skull was further imaged using a 48-megapixel sensor camera (Leica K3C camera on a Leica M80 stereoscope using LAS X software version 5.2.0.26130 (Leica Microsystems, Wetzlar, Germany)) to ensure fine detail and accuracy during collection and analysis. Images were taken from anterolateral and anteromedial angles to capture the entire orbit of each skull. Detailed views of the right and left orbits were used to identify the position of each foramen relative to recognizable landmarks. Images were uploaded and analyzed using ImageJ software (National Institutes of Health, Bethesda, MD, USA) to measure the diameter of all identified accessory foramina within each orbit. The resulting measurements were then compiled using Microsoft Excel (Microsoft Corporation, Redmond, WA, USA) and formatted into tables for analysis.

Statistical analysis

Due to the descriptive nature of this anatomical study, no inferential statistical tests were performed. Measurements of accessory foramina diameters obtained using ImageJ were compiled and organized in Microsoft Excel (Microsoft Corporation, Redmond, WA, USA). Data were summarized using descriptive statistics and presented in tabular form.

## Results

**Figure 1:**
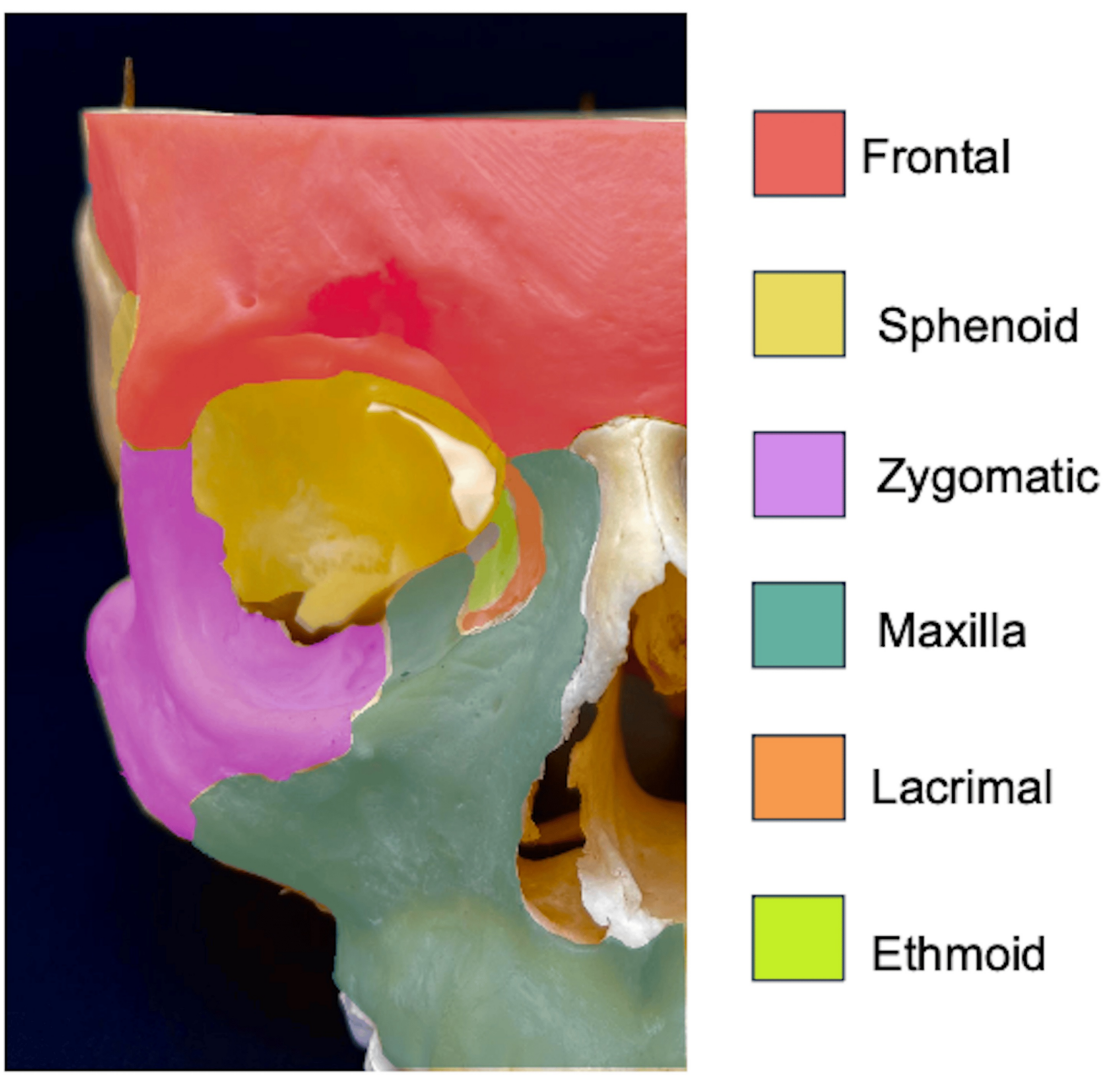
Orbital anatomy The orbit represents the intersection of multiple bones of the skull. The roof of the orbit is composed of the frontal and sphenoid bones. The lateral wall is composed of the frontal, sphenoid, and zygomatic bones. The floor is composed of the maxilla and zygomatic bone. The medial wall is composed of the frontal, sphenoid, lacrimal, and ethmoid bones. Image credit: Authors (formatted and edited in Microsoft PowerPoint (Microsoft® Corp., Redmond, WA))

The anatomy of the orbital region is depicted in Figure 1. The area of focus for the current study is the orbito-sphenoid region.

Ten cadaveric skulls of unknown name or origin were analyzed for the presence of accessory foramina in the orbito-sphenoid region. Our findings revealed three cases in which donor body skulls exhibited accessory foramina and generalized abnormalities. In two cases, the same skull had two accessory foramina in each orbit, while in the third case, an accessory foramen was observed only in the left orbit. The diameters of these accessory foramina ranged from 0.687 mm to 1.204 mm, with a mean of 1.015 mm. The remaining seven donor skulls showed no significant pathological findings or cranial abnormalities relevant to this study. These findings are summarized in Table 1.

**Table 1:** Anatomical variations in donor body skulls

Skull Identifier	Left / Right Orbit	General Findings	Accessory Foramen?	Locations	Diameter
3	Left	Accessory foramina present; irregular suture line; potential internal skull fracture on the lateral side of the left orbit	Yes: 2	Superior to the frontosphenoidal suture; directly on the frontosphenoidal suture	1.162 mm; 1.204 mm
3	Right	Accessory foramina present; suture line is irregular; pathological remodeling present	Yes: 2	Superior to the frontosphenoidal suture; directly on the frontosphenoidal suture	0.687 mm; 1.079 mm
10	Left	Accessory foramen present	Yes: 1	Medial and deep to the orbit	0.944 mm

**Figure 2:**
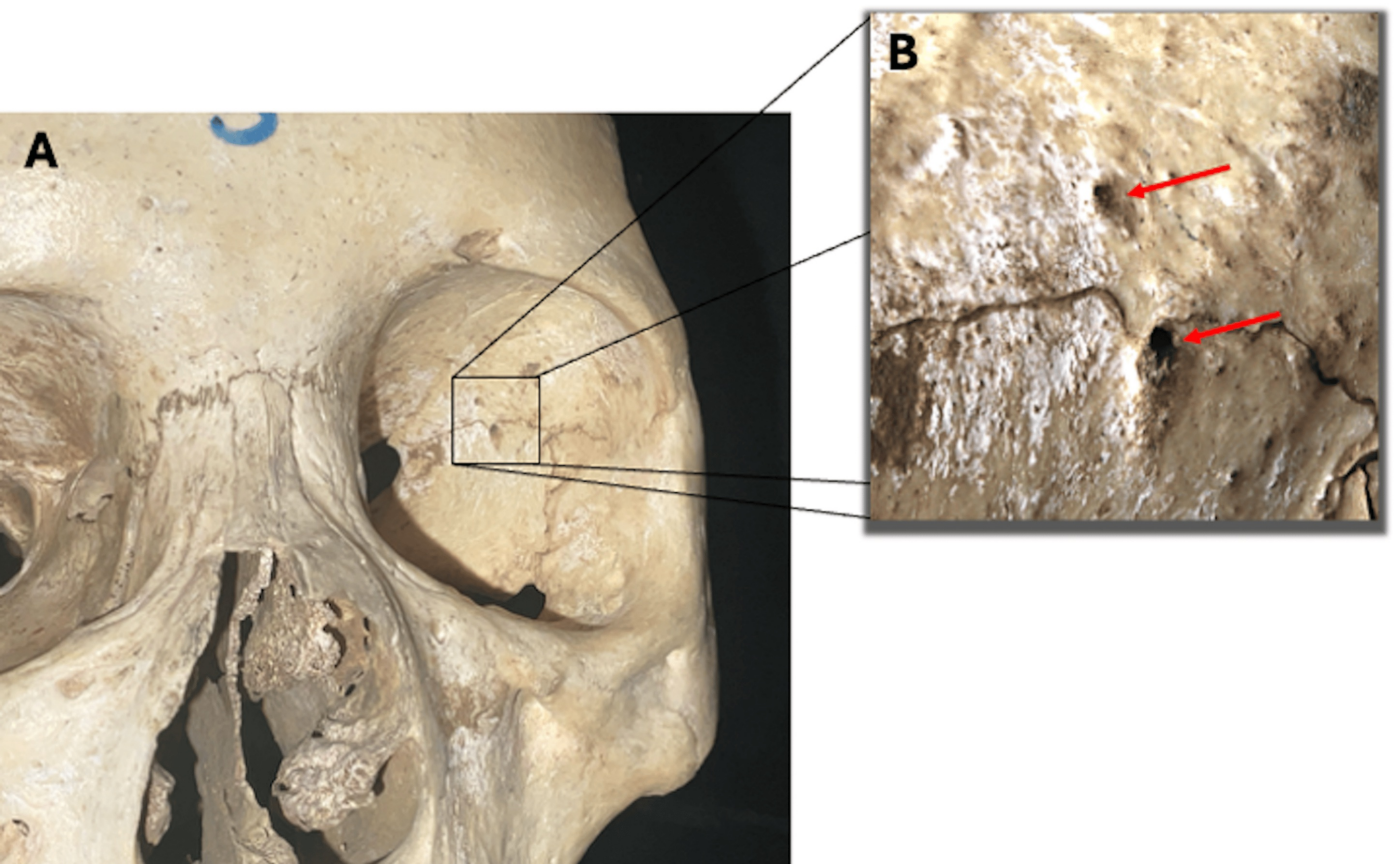
Two accessory foramina in the left orbit (A) Highlights the posterolateral wall of the left orbit of skull #3. In the zoomed-in image in (B), the red arrows point to the exact locations of the two accessory foramina found in this orbit. Image credit: Authors (formatted and edited in Microsoft PowerPoint (Microsoft® Corp., Redmond, WA))

Figure 2A depicts the left orbit of skull #3. The red arrows depict two accessory foramina in the zoomed-in Figure 2B. The foramen located superior to the frontosphenoidal suture has a diameter of 1.162 mm. The foramen located directly along the frontosphenoidal suture has a diameter of 1.204 mm.

**Figure 3:**
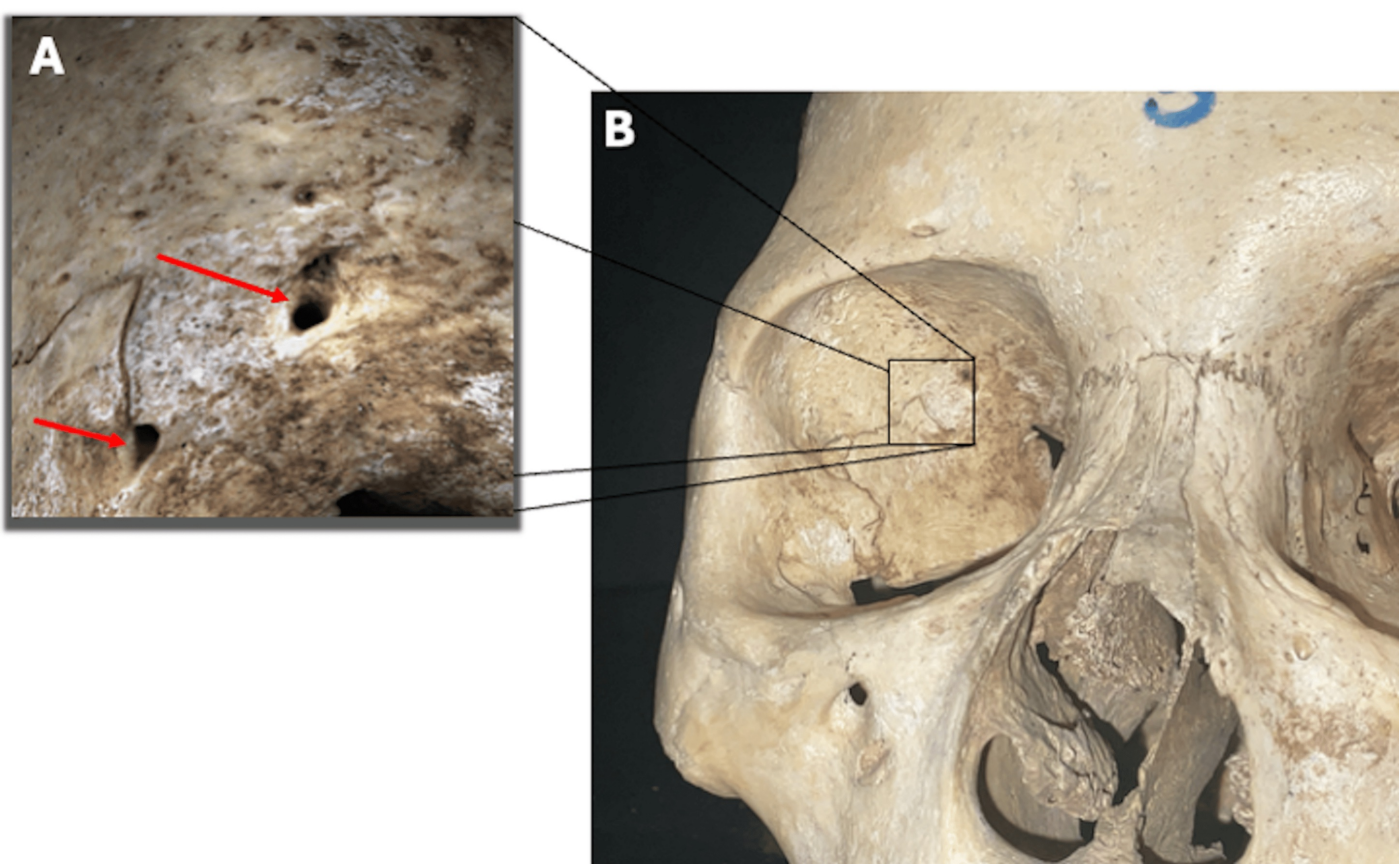
Two accessory foramina in the right orbit (A) Zoomed-in image showing the exact locations of the accessory foramina indicated by the red arrows. (B) Image of the posterolateral wall of the right orbit of skull #3. Image credit: Authors (formatted and edited in Microsoft PowerPoint (Microsoft® Corp., Redmond, WA))

Figure 3B depicts the right orbit of skull #3. The red arrows depict two accessory foramina in the zoomed-in Figure 3A. The foramen located superior to the frontosphenoidal suture has a diameter of 0.687 mm. The foramen located directly along the frontosphenoidal suture has a diameter of 1.079 mm.

**Figure 4:**
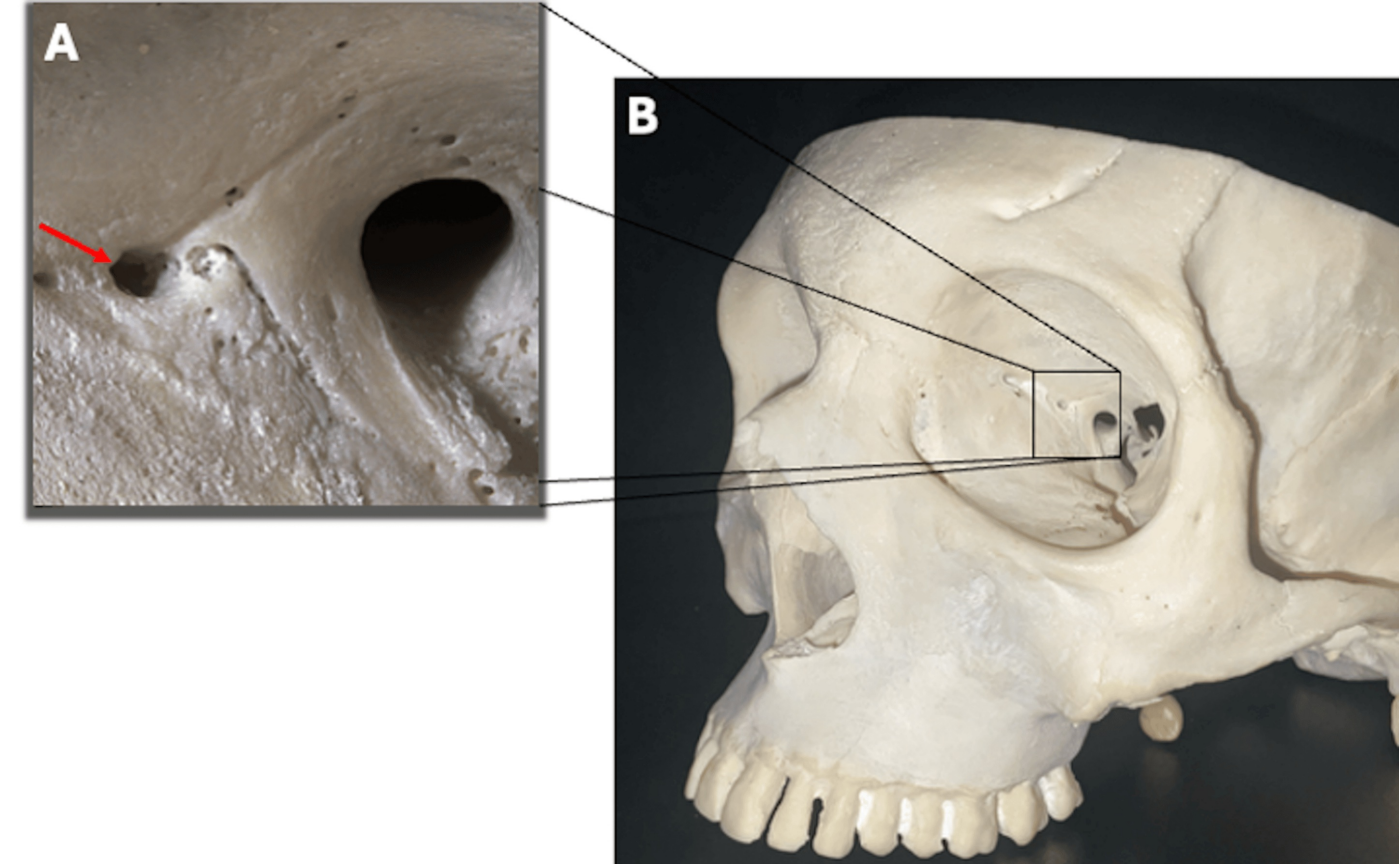
Single accessory foramen in the left orbit In the zoomed-in image (A), the red arrow points to the single accessory foramen found in this specimen. (B) Depicts a view highlighting the posteromedial wall of the left orbit of skull #10. Image credit: Authors (formatted and edited in Microsoft PowerPoint (Microsoft® Corp., Redmond, WA))

Figure 4B depicts the left orbit of skull #10. The red arrow depicts a single accessory foramen in the zoomed-in Figure 4A. The foramen is located deep to the lacrimal bone along the medial border of the orbit. Its diameter measures 0.944 mm.

## Discussion

Cranial variations

The findings of this study highlight the presence of variations within the orbit. These variations have the potential to directly influence surgical outcomes. More importantly, knowledge of their existence may prevent unnecessary complications when identified prior to surgery [12].

Perspectives on cranial remodeling

There are multiple perspectives on how cranial variations, particularly in bone, develop over time. One perspective focuses on embryological development: Between 23 and 26 days of gestation, the skull begins to form and undergoes significant remodeling to grow and strengthen in support of the cranial contents. During this process, the bones of the skull are connected by calvarial sutures, which prevent premature bone separation and allow for uniform remodeling [13]. Researchers suggest that the frequency of remodeling and the ability of these sutures to hold the skull bones together contribute to early variations in the embryo [14]. Within the orbit in particular, the sphenoid bone has a complicated embryologic origin, leaving plenty of opportunity for variation to arise [15]. While most of these variations are expected to be harmless due to the novel nature of the remodeling process, more pronounced variations can result in severe complications. Another perspective considers cranial trauma during childhood as a significant factor in the development of variations, given that the bones of the skull fuse at different times throughout an individual's life [11]. Despite differing viewpoints, there is a clear consensus that cranial remodeling results in variations such as accessory foramina, altered bone symmetry, accessory sutures, and neurovascular compression, all of which may lead to clinically significant outcomes.

Clinical significance

The findings of this study demonstrate both qualitative and quantitative clinical significance. Qualitatively, the visualization of accessory foramina within the orbit supports existing research on bone remodeling and highlights the common nature of cranial variations. Quantitatively, the measured diameters of these foramina establish a foundation for future studies to determine more accurate averages based on their location within the region. Despite the small sample size, the consistent presence of variations across multiple skulls suggests that cranial anomalies are not rare but instead quite common. This emphasizes the need for ongoing research with larger and more diverse populations to better understand these variations and their broader clinical implications.

Surgical implications

The anatomical variations observed in this study, particularly the accessory foramina in the orbits, carry significant implications for surgical planning and optimization, as they can impact surgical landmarks. For instance, descent of the infraorbital nerve into the maxillary sinus has been shown to position the infraorbital foramen farther from the inferior orbital rim, increasing the risk of nerve injury if unrecognized [16]. Additionally, when performing an orbital decompression to treat thyroid eye disease, the surgeon must break and remove the medial aspect of the orbital floor while taking care not to damage the infraorbital nerve [17]. If the patient had a varied neurovasculature, this precise surgery could have significant consequences. Other procedures involving the orbit, such as radiofrequency rhizotomy for the treatment of trigeminal neuralgia and tumor resections, require similar precision and accuracy to prevent iatrogenic injury [11]. Even in less invasive operations, nerve structures taking alternate paths through the orbit can lead to incomplete anesthesia. These variations may still provide normal function, but can lead to less successful outcomes if the structures are not properly localized. For this reason, specific knowledge of anatomical arrangement prior to operating is essential.

Limitations

One limitation of this study is the small sample size, as only 10 skulls were examined to identify accessory foramina. Additionally, all specimens were collected from donor bodies in the Midwestern United States, which limits the generalizability of these findings. Another limitation is the lack of demographic and medical data for these donor bodies, including race, sex, medical history, and cause of death. As a result, potential correlations between these factors and the presence of accessory foramina could not be evaluated.

Future directions

To enhance the generalizability and accuracy of our study, we aim to increase the sample size and gather donor demographics (age, sex, and ethnicity), as well as cause of death, to facilitate additional potential correlations.

## Conclusions

The donor body skulls analyzed in this study provided valuable insights into the existence of variations within the sphenoid bone of the orbit. Historical studies have shown anatomical variations, such as additional accessory foramina, can increase the risk of neurovascular injury, potentially leading to irreparable damage. Our study supports findings from previous research demonstrating variation in the cranium through the presence of accessory foramina in the orbit. We plan to continue data collection for the study to increase the accuracy and generalizability of the study to a larger population. We hope our findings encourage a more cautious approach to surgical planning and highlight the importance of pre-surgical visualization. As with all anatomical variations, cranial variations are common and, while unexpected in practice, should be carefully considered for every patient to optimize surgical success.
